# Sublingual Immunization of Trivalent Human Papillomavirus DNA Vaccine in Baculovirus Nanovector for Protection against Vaginal Challenge

**DOI:** 10.1371/journal.pone.0119408

**Published:** 2015-03-19

**Authors:** Hee-Jung Lee, Hansam Cho, Mi-Gyeong Kim, Yoon-Ki Heo, Yeondong Cho, Yong-Dae Gwon, Ki Hoon Park, Hyerim Jin, Jinyoung Kim, Yu-Kyoung Oh, Young Bong Kim

**Affiliations:** 1 Department of Bio-industrial Technologies, Konkuk University, Seoul, Republic of Korea; 2 College of Pharmacy and Research Institute of Pharmaceutical Sciences, Seoul National University, Seoul, Republic of Korea; Shanghai Medical College, Fudan University, CHINA

## Abstract

Here, we report the immunogenicity of a sublingually delivered, trivalent human papillomavirus (HPV) DNA vaccine encapsidated in a human endogenous retrovirus (HERV) envelope-coated, nonreplicable, baculovirus nanovector. The HERV envelope-coated, nonreplicable, baculovirus-based DNA vaccine, encoding HPV16L1, -18L1 and -58L1 (AcHERV-triHPV), was constructed and sublingually administered to mice without adjuvant. Following sublingual (SL) administration, AcHERV-triHPV was absorbed and distributed throughout the body. At 15 minutes and 1 day post-dose, the distribution of AcHERV-triHPV to the lung was higher than that to other tissues. At 30 days post-dose, the levels of AcHERV-triHPV had diminished throughout the body. Six weeks after the first of three doses, 1×10^8^ copies of SL AcHERV-triHPV induced HPV type-specific serum IgG and neutralizing antibodies to a degree comparable to that of IM immunization with 1×10^9^ copies. AcHERV-triHPV induced HPV type-specific vaginal IgA titers in a dose-dependent manner. SL immunization with 1×10^10^ copies of AcHERV-triHPV induced Th1 and Th2 cellular responses comparable to IM immunization with 1×10^9^ copies. Molecular imaging revealed that SL AcHERV-triHPV in mice provided complete protection against vaginal challenge with HPV16, HPV18, and HPV58 pseudoviruses. These results support the potential of SL immunization using multivalent DNA vaccine in baculovirus nanovector for induction of mucosal, systemic, and cellular immune responses.

## Introduction

Needle-free vaccination via mucosal routes has drawn increasing recent attention as a vaccine delivery strategy. An ideal vaccine against an infectious pathogen should prime the host for induction of pathogen-specific memory immune responses at the appropriate mucosal compartment, thereby preventing the entry and/or replication of the invading pathogen at the site of infection [1]. Mucosal immunizations via nasal, buccal, or sublingual routes have recently emerged as alternatives to intramuscular (IM) vaccine administration. Non-parenteral, needle-free mucosal vaccination has several advantages, including reduced pain stresses, costs, and viral transmission associated with the injection [2,3].

Current studies have established that sublingual (SL) immunization can efficiently stimulate mucosal immunity and induce systemic humoral immune and cytotoxic T lymphocyte (CTL) responses [4,5]. In recent years, a number of studies have explored the potential of SL immunization in eliciting desired immune responses against various potential vaccine components, including protein antigens [6,7], and live-attenuated viruses [8,9]. However, few studies have investigated SL delivery of DNA vaccines using viral vectors.

In a previous study, we constructed a human endogenous retrovirus (HERV) envelope-coated, nonreplicable, baculovirus-based DNA vaccine against human papillomavirus (AcHERV-HPV). IM administration of AcHERV-based monovalent HPV16L1 [10], bivalent HPV16L1 and -18L1 [11], or trivalent HPV16L1, -18L1, and -58L1 (AcHERV-triHPV) [12] gene constructs all induced high levels of humoral and cellular immunogenicity and provided complete protection against HPV type-specific pseudoviruses (PVs). Here, we tested whether a DNA vaccine encapsidated in this AcHERV system could be delivered via the SL route by administering AcHERV-triHPV in mice sublingually without any adjuvant. Here, we report the immunogenicity of AcHERV-triHPV following SL immunization.

## Materials and Methods

### Generation of AcHERV-triHPV

AcHERV-triHPV was produced using a Bac-to-Bac baculovirus expression system (Invitrogen, CA, USA) according to the manufacturer’s instructions [12]. Briefly, the recombinant baculovirus was constructed to encode a codon-optimized envelope gene of human endogenous retrovirus (HERV; GenBank accession number NM014590; GenScript Corp., Piscataway, NJ, USA) and sequences of the three HPV genes, 16L1, 18 L1, and 58 L1 (kindly supplied by Dr. Schiller, National Cancer Institute, National Institutes of Health, USA) under the control of the human elongation factor1α promoter. *Spodoptera frugiperda* 9 (Sf9) cells were from Invitrogen (Catalog No. 11496–015), and cultured at 28°C in Sf-900 II medium (Invitrogen) supplemented with 100 units/ml of Gibco antibiotic-antimycotic (Invitrogen). AcHERV-triHPV was amplified by propagation in Sf9 cells and purified by first centrifuging at 2,000×g at 4°C for 10 minutes to remove virus-infected cell debris. Thereafter, supernatants were overlaid on a 30% sucrose cushion and centrifuged at 35,000 rpm at 4°C for 1.5 hour in a 50.2Ti rotor (Beckman Coulter Inc., CA, USA). The pellet was re-suspended in phosphate-buffered saline (PBS; Invitrogen) and used for immunization.

### Animals

Six-week-old female BALB/c mice were purchased from Orient-Bio (Seungnam, Kyonggi-do, Republic of Korea) and housed in filter-top cages, with water and food provided ad libitum. Mice were maintained in accordance with the Guide for the Care and Use of Laboratory Animals of Konkuk University (Seoul, Republic of Korea), and were housed in a Bio-safety Level 2 facility. The use of animals in these experiments was approved by the Institutional Animal Care and Use Committee of Konkuk University (Approval No. KU12078). Throughout the study, the condition of animals was monitored twice a day. In this study, no mice exhibited symptoms of illness or appeared to be close to death. Moreover, no mice died during the monitoring phase. After final monitoring, mice were humanely euthanized using cervical dislocation according to the AVMA guidelines for the euthanasia of animals.

### Biodistribution studies

For biodistribution studies, AcHERV-triHPV was sublingually administered, and the levels of AcHERV-triHPV in various tissues were measured using quantitative real-time polymerase chain reaction (qRT-PCR). Mice were anesthetized with 40 mg/kg of Zoletil 50 (Virbac Laboratories, Carros, France) and 5 mg/kg of Rompun (Bayer Korea, Seoul, Republic of Korea). Mice were sublingually administered 1×10^9^ copies of AcHERV-triHPV using a previously reported procedure [13]. Mice were sacrificed by CO_2_ inhalation at various time points, and tissues were collected. Total blood and tissue DNA was extracted using a DNeasy Tissue Kit (Qiagen, Valencia, CA, USA), as described by the manufacturer. For qRT-PCR, 10-fold serially diluted plasmids encoding HPV16L1, -18L1 and -58L1 (pFB-HERV-HPV16–58–18L1), encompassing a concentration range of 1×10^2^ to 1×10^8^ copies/μl, were used to construct a standard curve. The levels of the HPV16–58–18L1 gene in mouse samples were determined by qPCR amplification of a genomic DNA (gDNA) template (200 ng) using LightCycler FastStart DNA Master SYBR Green I (Roche Diagnostics Gmbh, Mannheim, Germany). The amplification conditions consisted of an initial denaturation step at 94°C for 5 minutes, followed by 40 cycles of 30 seconds at 94°C, 20 seconds at 62°C, and 20 seconds at 72°C. The primers for HPV16L1 detection were 5′-CAG CGA GAC CAC CTA CAA GA-3′ (forward primer) and 5′-GCT GTT CAT GCT GTG GAT GT-3′ (reverse primer), generating a 139-bp product. Amplification of glyceraldehyde phosphate dehydrogenase (GAPDH) mRNA was used to control for the efficiency of qRT-PCR among samples.

### SL and IM immunization of mice

**Fig 1:**
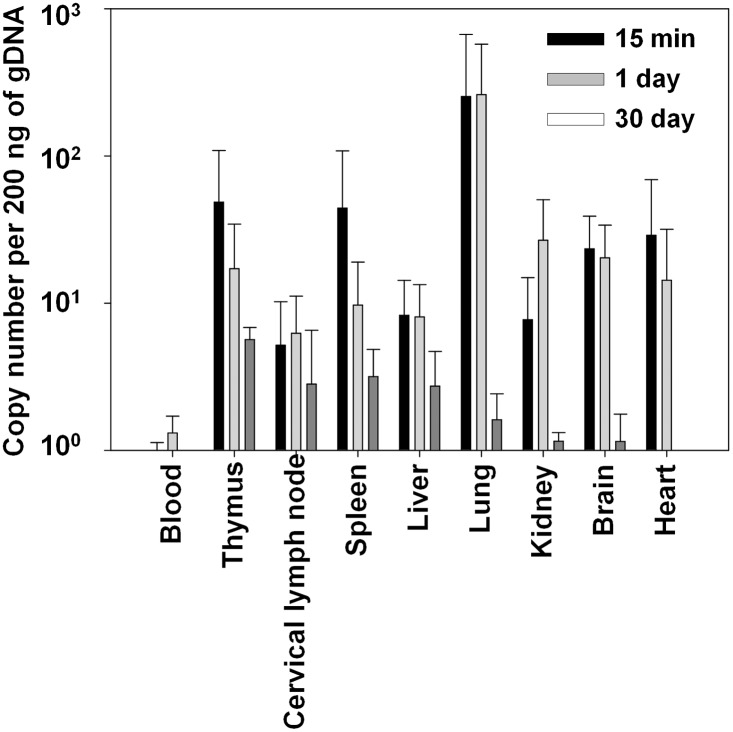
AcHERV-triHPV levels after SL administration. All mice were given a single dose (1×10^9^ copies) of AcHERV-triHPV via the SL route. Blood and tissue samples were collected 15 minutes, 1 day, and 30 days after immunization. AcHERV-triHPV copy numbers were measured using qRT-PCR. Data are presented as means ± SD (n = 5).

Mice were immunized with AcHERV-triHPV vaccines via the SL or IM route as depicted in Fig. 1. All mice received three immunizing doses at 2-week intervals by the same route. For SL immunization, mice were anesthetized with 40 mg/kg of Zoletil 50 (Virbac Laboratories) and 5 mg/kg of Rompun (Bayer Korea). Mice received different doses of sublingually delivered AcHERV-triHPV using a previously described procedure [13]. To prevent swallowing during SL immunization, the total volume of the inoculum was limited to 15 μl/mouse. For IM immunization, mice were intramuscularly injected in the hind legs with 1×10^9^ copies of AcHERV-triHPV. For comparison, mice were immunized three times with Cervarix (GlaxoSmithKline, Middlesex, UK) at 1/20^th^ of a human dose.

Serum and vaginal samples were collected 2, 4, and 6 weeks after the first immunization (day 0) via the SL or IM route (Fig. 1). Serum samples were obtained by centrifugation of whole blood collected from the right external jugular vein. Vaginal secretion samples were collected by rinsing the vaginal cavity five times with 30 μl of PBS. Vaginal secretion samples were then microcentrifuged at 13,000 rpm for 10 minutes, and the supernatants were collected and stored at -80°C until analyzed.

### Production of HPV type-specific PVs

All HPV type-specific PVs were produced as previously described [14–16]. The capsid-encoding plasmids p16L1/L2, p18L1/L2, and p58L1/L2 were used to produce HPV16 PV, HPV18 PV, and HPV58 PV, respectively. For neutralization assays, pYSEAP (a secreted alkaline phosphatase; SEAP expression plasmid) was encapsidated. For *in vivo* imaging, the plasmid pCLucf (a firefly luciferase expression plasmid) was encapsidated [17]. All plasmids used for *in vitro* and *in vivo* assays were prepared as described on the National Cancer Institute website (http://home.ccr.cancer.gov/lco/default.asp).

### Measurement of anti-HPV16L1, -18L1, and -58L1 antibodies

The levels of antibodies specific for HPV16L1-, -18L1, and -58L1 were measured by enzyme-linked immunosorbent assay (ELISA) as preciously described [12]. Briefly, ELISA plates were coated with 1 μg/ml of HPV16, -18, or -58 PVs. After incubation for 16 hours at 4°C, the plates were washed and blocked with 2% (w/v) bovine serum albumin. Serially diluted mouse sera or vaginal secretion samples were added and incubated for 2 hours at room temperature. After washing, the plates were incubated with peroxidase-conjugated goat anti-mouse IgG antibody (1:2000; Santa Cruz Biotechnology, Santa Cruz, CA, USA) or goat anti-mouse IgA antibody (1:1000; Santa Cruz Biotechnology) for 1 hour at 37°C. For color development, 1-Step Turbo TMB (3,3′,5,5′-tetramethyl benzidine substrate solution, Pierce, USA) was added. The reaction was stopped by adding 1N H_2_SO_4_, and the absorbance was measured at 450 nm. Endpoint titers were defined as the highest serum dilutions that resulted in an absorbance for non-immunized serum that reached a cutoff value, and were expressed as the group geometric means ± SD.

### *In vitro* neutralization of HPV PVs

Neutralization assays were performed using SEAP-expressing HPV16 PVs according to a previously described method [14]. Briefly, OptiPrep-purified SEAP HPV16, -18, and -58 PVs were diluted 3,000-fold and incubated on ice for 1 hour with 3-fold serial dilutions of serum. 293TT cells were infected by incubating with each PV–antibody mixture for 72 hours. The SEAP content in 10 μl of clarified cell supernatant was determined using the Great EscAPe SEAP Chemiluminescence Kit (Clontech, Mountain View, CA, USA). Neutralization titers were defined as the reciprocal of the highest serum dilution that caused at least a 50% reduction in SEAP activity.

### Enzyme-linked immunospot assay for interferon-γ and interleukin-4

The induction of HPV16L1-, HPV18L1-, and HPV58L1-specific CD8^+^ T cells was determined by measuring the production of interferon-γ (IFN-γ) and interleukin-4 (IL-4) using an enzyme-linked immunospot (ELISPOT) assay. Two weeks after the final immunization with AcHERV-triHPV, splenocytes were isolated from immunized mice. The ELISPOT plate (ELISPOT kit; BD Biosciences, San Jose, CA, USA) was coated with 0.2 μg of anti-mouse IFN-*γ* or IL-4 capture antibody. Plates were blocked by incubating with 10% fetal bovine serum at 37°C, and seeded with splenocytes at 1×10^6^ cells per well in 100 μl of medium. For stimulation, 1×10^8^ copies of HPV16, -18, or -58 PVs were added and plates were incubated for 24 hours at 37°C. Plates were then washed with PBS containing 0.05% Tween-20 and treated with 20 ng of biotinylated anti-mouse IFN-*γ* or IL-4 detection antibody. After 2 hours, streptavidin-alkaline phosphatase was added, and color was developed using an aminoethylcarbazole (AEC) substrate reagent (BD Biosciences). The number of spots was counted using an ELISPOT reader (AID Elispot Reader ver. 4; AID Gmbh., Straßburg, Germany).

### Mouse model of vaginal HPV PV infection

Three weeks after the final immunization with AcHERV-triHPV, mice were challenged with HPV PVs as described previously [18,19]. Seven days before the *in vivo* genital challenge with PVs, mice were synchronized in a diestrus-like status by subcutaneous injection of 3 mg DepoProvera (Pfizer AG, Zurich, Switzerland). Six hours prior to each PV challenge, deeply anesthetized mice were intravaginally pretreated with 20 μl of 4% nonoxynol-9 (Sigma, St. Louis, MO, USA). Mice were genitally challenged with 5×10^8^ copies of HPV16 PV, HPV18 PV, or HPV58 PV, each in a 20-μl solution containing 2% carboxymethylcellulose (Sigma). HPV infection was monitored by measuring luciferase expression in the genital tract on day 3 post-challenge. On the final day, anesthetized mice were instilled intraperitoneally with 0.3 mg (in 30 μl PBS) of luciferin (Caliper Life Sciences, Hopkinton, MA, USA). Luciferase expression was visualized as light emission measured in molecular images acquired for 10 minutes using an IVIS 200 bioluminescence imaging system (Xenogen, Cranbury, NJ, USA). Equal areas encompassing the site of virus inoculation were analyzed using Living Image 2.20 software (Xenogen).

### Statistical analysis

All data were analyzed by analysis of variance (ANOVA) and post hoc Student-Newman-Keuls tests using SigmaStat software (Systat Software, San Jose, CA, USA). P-values less than 0.05 were considered significant.

## Results

### Biodistribution of AcHERV-triHPV following SL administration

Sublingually administered AcHERV-triHPV was absorbed and distributed throughout the body (Fig. 1). AcHERV-triHPV was detected in blood, thymus, cervical lymph node, spleen, liver, lung, kidney, brain, and heart at 15 minutes post-dose. Thymus, spleen, lung, brain, and heart showed levels greater than 10 copies per 200 ng of gDNA at 15 minutes post-dose. One day after SL immunization, distribution to the spleen was decreased to less than 10 copies per 200 ng of gDNA, and the distribution to the kidney was increased to greater than 10 copies per 200 ng of gDNA. At 15 minutes and 1 day after SL administration, the highest distribution of AcHERV-triHPV was observed in the lung (Fig. 1). The levels of AcHERV-triHPV decreased throughout the body over time. Thirty days after SL administration, no tissues showed levels of AcHERV-triHPV greater than 10 copies per 200 ng of gDNA.

### Humoral immune responses to sublingually administered AcHERV-triHPV

**Fig 2:**
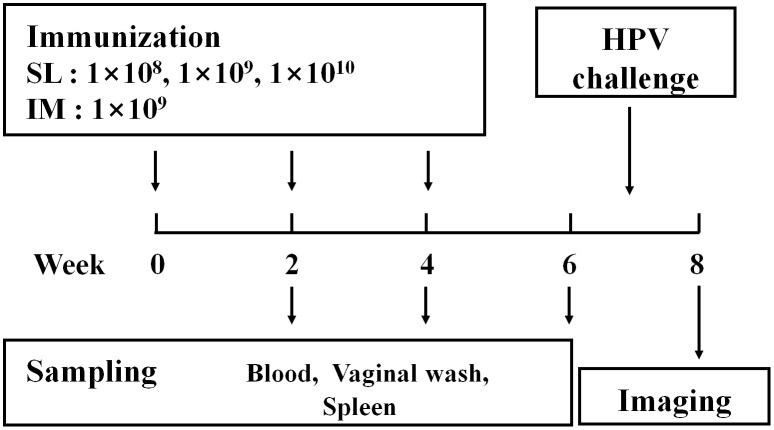
SL and IM immunization and sampling schedules. All mice were given three immunizations at 2-week intervals via the SL or IM route. Serum and vaginal secretion samples were collected 2, 4, and 6 weeks after the first immunization. Spleens were collected 6 weeks after the first immunization.

**Fig 3:**
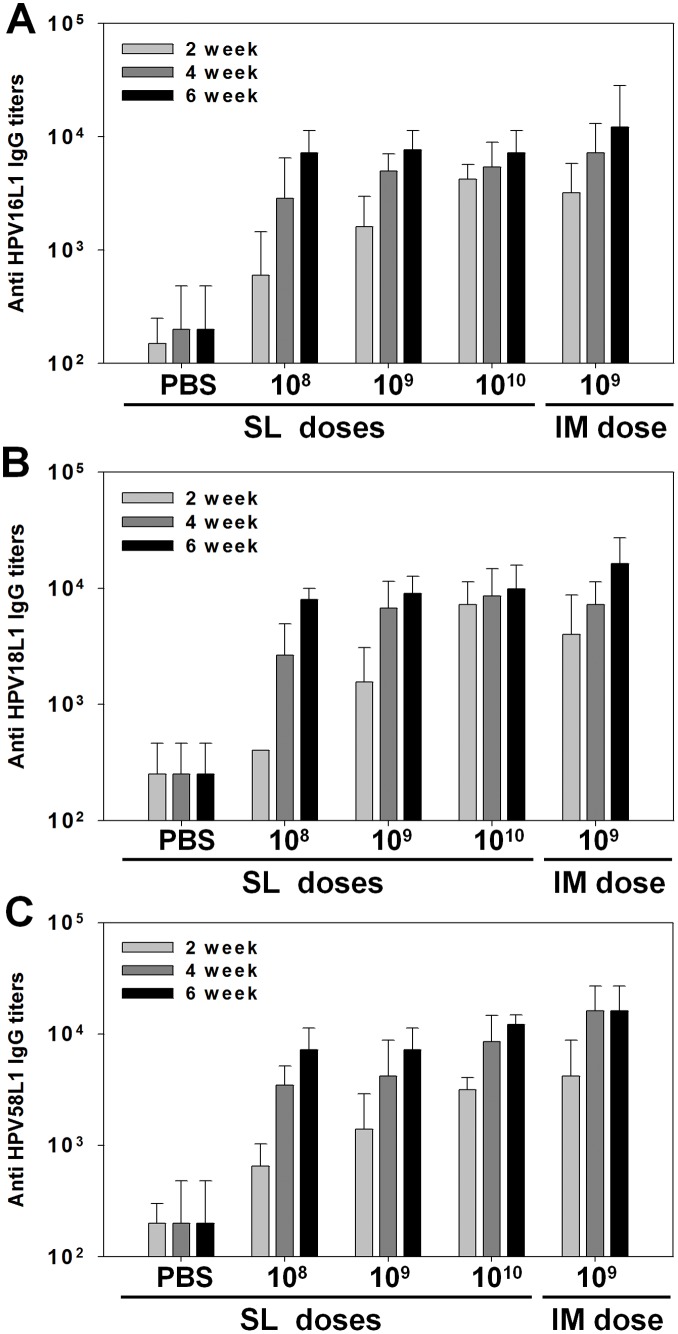
HPV type-specific serum IgG antibody titers after SL or IM immunization. BALB/c mice were administered AcHERV-triHPV or PBS (control) three times over a 2-week interval via the SL or IM route. Mice were sublingually immunized with three different doses of AcHERV-triHPV (1×10^8^, 1×10^9^, and 1×10^10^ copies per mouse) or were immunized intramuscularly with 1×10^9^ copies per mouse. Samples were collected 2, 4, and 6 weeks after the first immunization. Antigen-specific serum IgG antibody titers against HPV16L1 (A), HPV18L1 (B), and HPV58L1 (C) were determined by ELISA. *P < 0.05 compared with other groups (ANOVA and Student-Newman-Keuls test).

AcHERV-triHPV was administered three times in SL or IM route. The injection and sampling scheme is shown in Fig. 2. SL immunization with AcHERV-triHPV induced L1 type-specific humoral immune responses comparable to those observed following IM immunization, inducing serum IgG antibodies against HPV16L1 (Fig. 3A), HPV18L1 (Fig. 3B), and HPV58L1 (Fig. 3C). SL administration of AcHERV-triHPV induced a dose-dependent increase in serum IgG antibodies over a range of 1×10^8^ to 1×10^10^ copies that was clearly observed 2 and 4 weeks after the first immunization. However, this dose-dependence of serum IgG antibody production disappeared by 6 weeks after the first SL immunization. Moreover, at 6 weeks after the first immunization, the levels of serum IgG were not significantly different between SL and IM immunization. Notably, the induction of serum IgG against HPV16L1, -18L1, and -58L1 by SL immunization with 1×10^8^ copies of AcHERV-triHPV was comparable to that induced by IM immunization with 1×10^9^ copies at 6 weeks after the first immunization.

**Fig 4:**
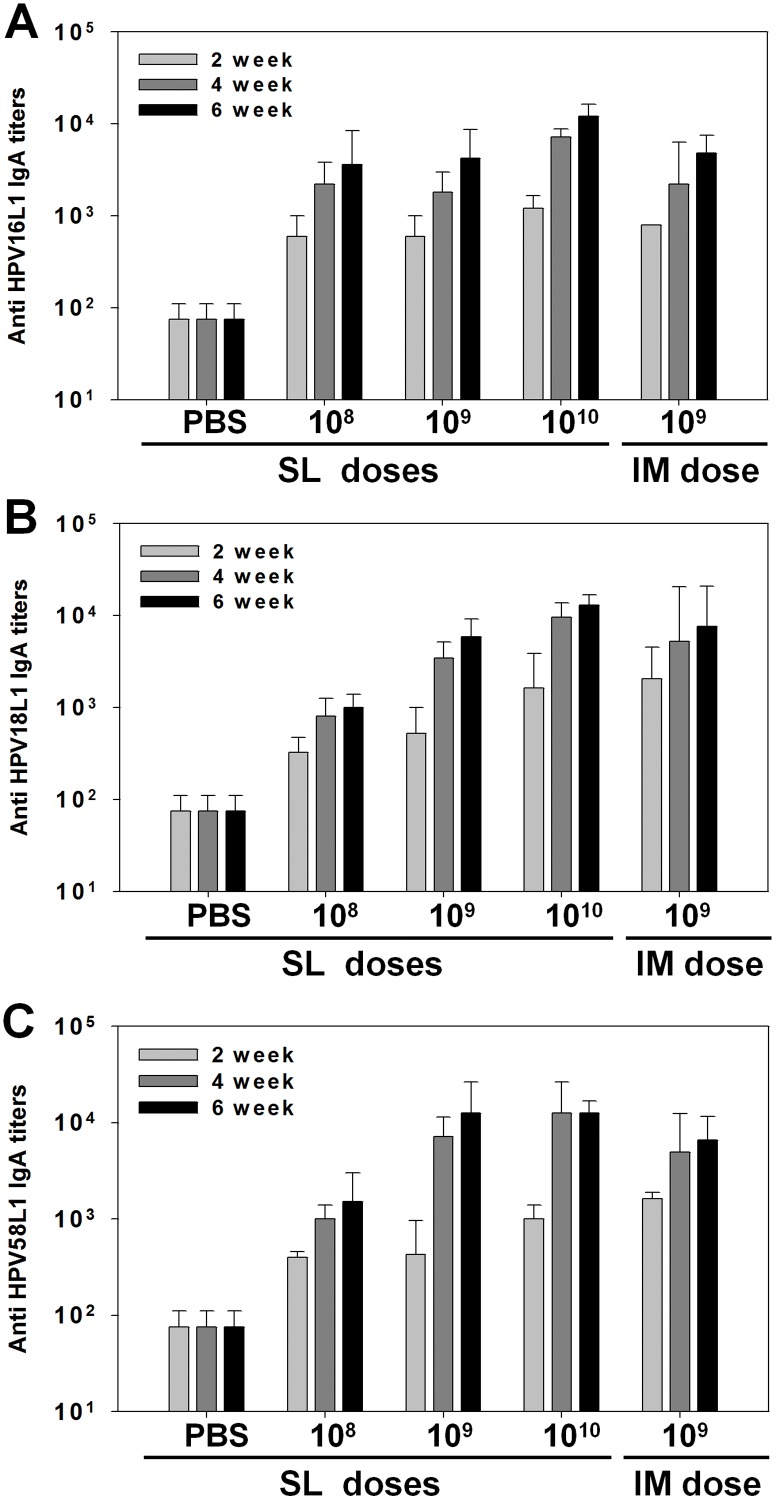
HPV type-specific vaginal IgA antibody titers after SL or IM immunization. Mice were administered AcHERV-triHPV or PBS (control) three times over a 2-week interval by the SL or IM route. Mice were sublingually immunized with three different doses of AcHERV-triHPV (1×10^8^, 1×10^9^, and 1×10^10^ copies per mouse) or immunized intramuscularly with 1×10^9^ copies per mouse. Vaginal secretion was sampled 2, 4, and 6 weeks after the first immunization. Antigen-specific vaginal IgA antibody titers against HPV16L1 (A), HPV18L1 (B), and HPV58L1 (C) were determined by ELISA. *P < 0.05 compared with other groups (ANOVA and Student-Newman-Keuls test).

Although serum IgG induction after SL immunization showed little sustained dose dependence, vaginal IgA levels increased in a dose-dependent manner by SL immunization. AcHERV-triHPV induced vaginal IgA against HPV16L1 (Fig. 4A), HPV18L1 (Fig. 4B), and HPV58L1 (Fig. 4C). For all three types of IgA, the highest IgA antibody titers were observed after SL immunization with the highest dose (1×10^10^ copies). Six weeks after the first immunization, the levels of vaginal IgA following SL immunization with 1×10^9^ copies were comparable to those following IM immunization with the same dose.

### Induction of neutralizing antibodies by sublingually administered AcHERV-triHPV

**Fig 5:**
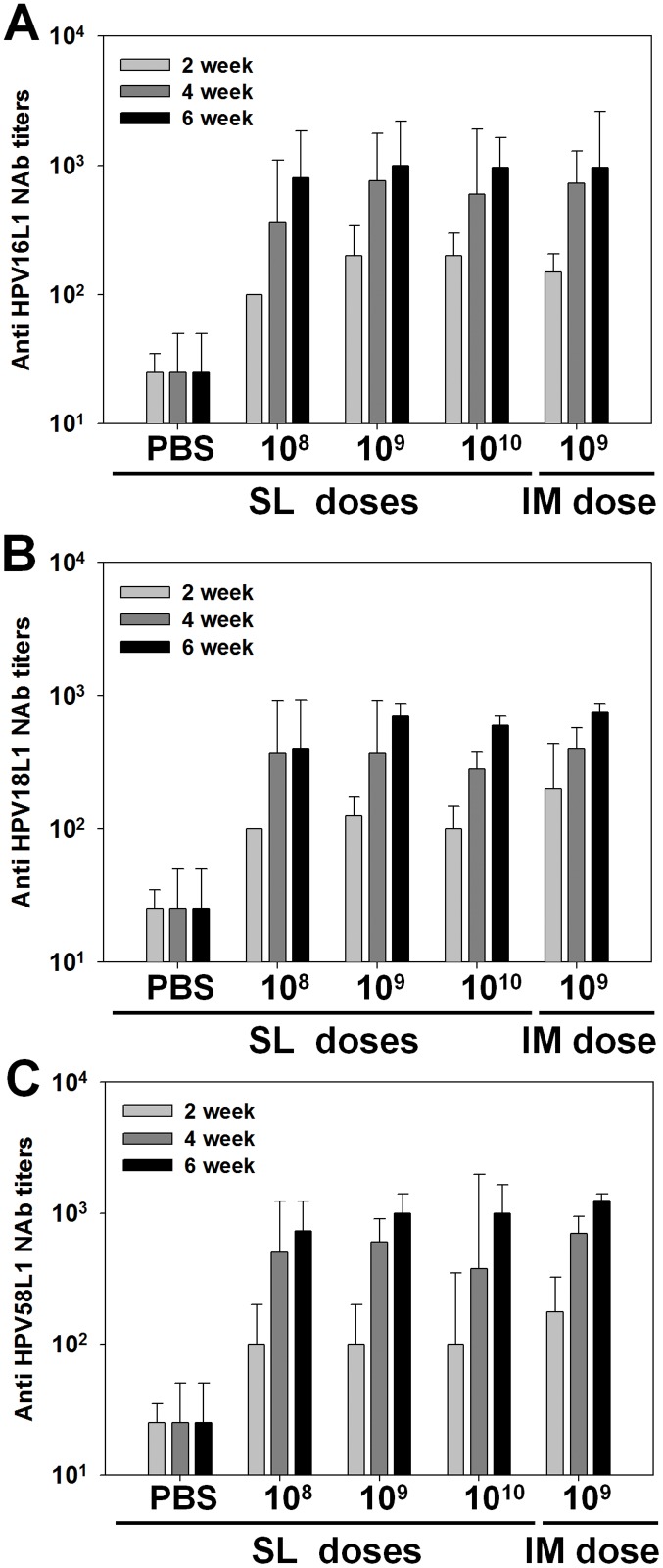
Induction of HPV type-specific neutralizing antibodies following SL or IM immunization. Mice were administered AcHERV-triHPV or PBS (control) three times over a 2-week interval by the SL or IM route. Mice were sublingually immunized with three different doses of AcHERV-triHPV (1×10^8^, 1×10^9^, and 1×10^10^ copies per mouse) or immunized intramuscularly with 1×10^9^ copies per mouse. Antigen-specific neutralizing antibody titers against HPV16L1 (A), HPV18L1 (B), and HPV58L1 (C) were determined by SEAP assay. Neutralization assays were performed using serially diluted mouse sera and HPV16, -18, or -58 PVs. Data are expressed as geometric means (log) of reciprocal serum dilutions that yielded a 50% reduction in SEAP (n = 5).

SL administration of AcHERV-triHPV induced type-specific neutralizing antibodies against HPV16L1 PVs (Fig. 5A), HPV18L1 PVs (Fig. 5B), and HPV58L1 PVs (Fig. 5C). Similar to serum IgG antibodies (Fig. 3), serum-neutralizing antibodies were induced in a dose-dependent manner by sublingually administered AcHERV-triHPV 2 and 4 weeks after the first administration, but neutralizing antibody titers were not significantly different among the three doses 6 weeks after the first immunization. Consistent with its ability to induce serum IgG (Fig. 3), SL immunization with 1×10^8^ copies of AcHERV-triHPV induced neutralizing antibodies against HPV16L1, -18L1, and -58L1 6 weeks after the first immunization to a degree comparable to that of IM immunization with 1×10^9^ copies.

### HPV type-specific Th1 and Th2 cell responses to sublingually administered AcHERV-triHPV

**Fig 6:**
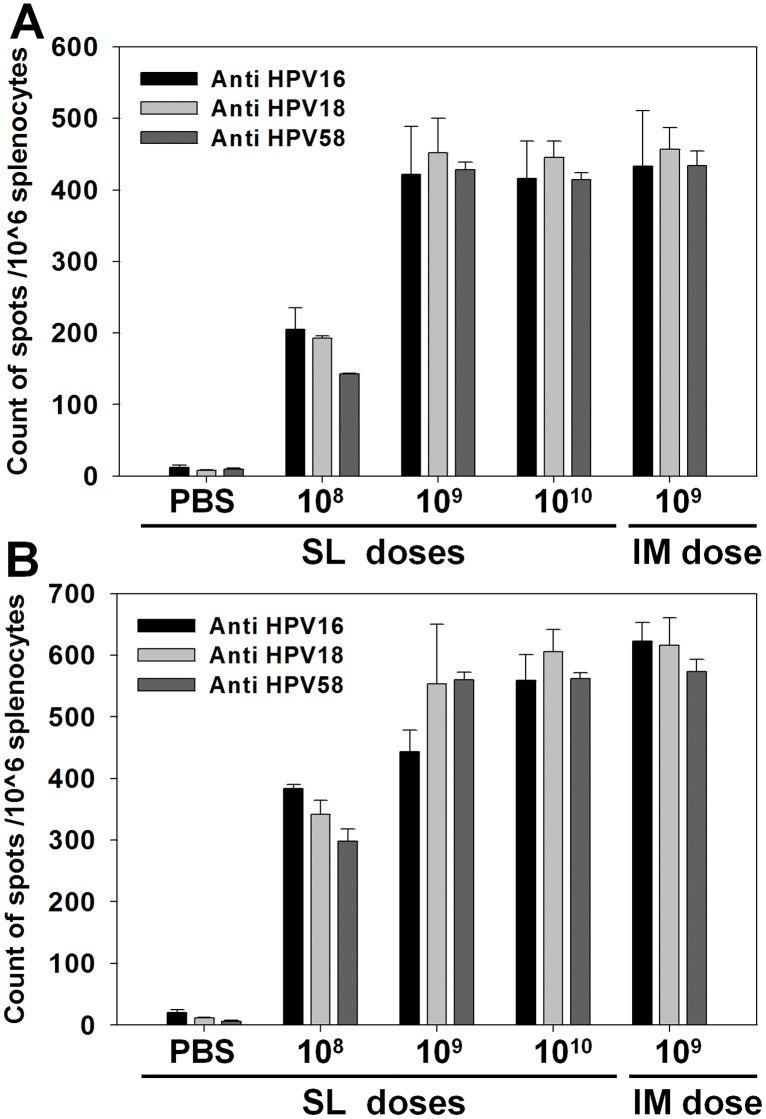
Th1 and Th2 cytokine induction following SL or IM immunization. Mouse splenocytes were harvested 6 weeks after the first immunization. ELISPOT assays were used to determine the number of (A) IFN-γ-producing HPV16-, HPV18-, or HPV58-specific CD8^+^ T cells, and (B) IL-4-producing HPV16-, HPV18-, or HPV58-specific CD4^+^ T cells. Values represent the number of spots per 10^6^ splenocytes following stimulation with HPV16, -18 or -58 PVs (PV16, PV18, or PV58).

SL immunization with three doses of AcHERV-triHPV induced both Th1 and Th2 cell responses in an HPV type-specific and dose-dependent manner. Splenic T cell responses were detected using IFN-γ and IL-4 ELISPOT assays. SL immunization with AcHERV-triHPV increased the production of IFN-γ (Fig. 6A) and IL-4 (Fig. 6B) by splenic T cells after stimulation with HPV16L1, -18L1, or -58L1 PVs. Although serum IgG production (Fig. 3) and neutralizing antibody induction (Fig. 5) showed little dependence on the dose of sublingually administered AcHERV-triHPV, cellular immune responses were dose dependent. Following SL immunization, 1×10^9^ and 1×10^10^ copies of AcHERV-triHPV produced at least 2-fold higher production of IFN-γ regardless of the stimulating HPV type compared to 1×10^8^ copies (Fig. 6A). In terms of Th2 cell responses, 1×10^9^ and 1×10^10^ copies of sublingually administered AcHERV-triHPV induced at least 1.6-fold higher production of IL-4 than 1×10^8^ copies except in the case of stimulation with HPV16L1 PVs (Fig. 6B). SL immunization with 1×10^10^ copies of AcHERV-triHPV induced Th1 and Th2 cellular responses comparable to those induced by IM immunization with 1×10^9^ copies.

### Protection against HPV PV challenge in mice by SL AcHERV-triHPV

**Fig 7:**
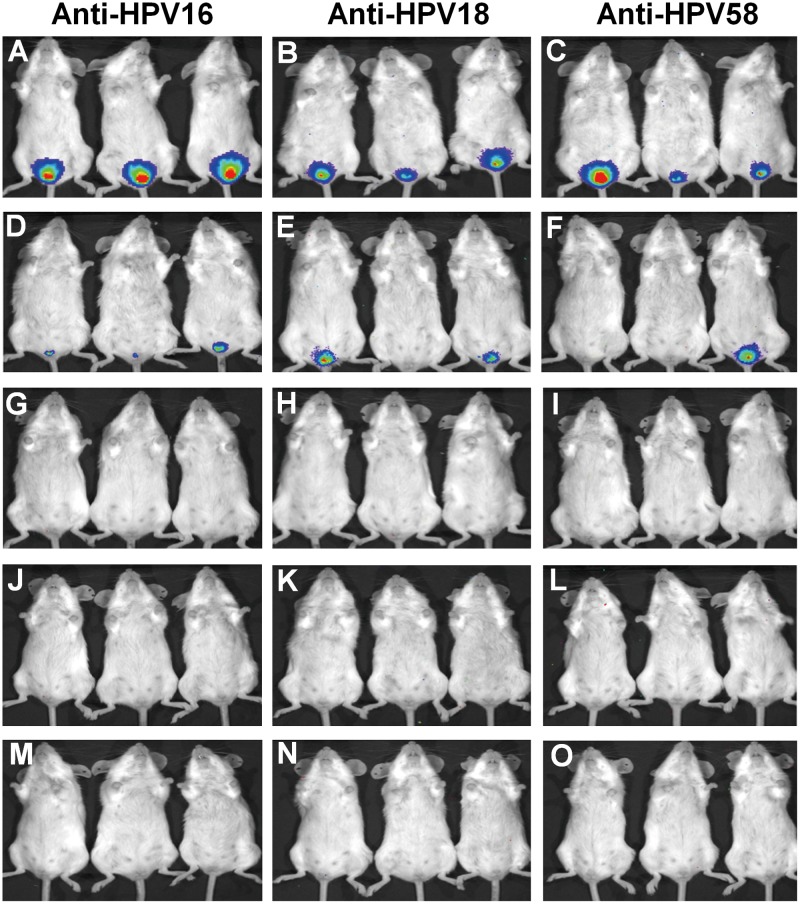
Vaginal challenge of AcHERV-triHPV-immunized mice with PVs. Mice were administered AcHERV-triHPV or PBS (control) three times over a 2-week interval by the SL or IM route. (A-C) Mice sublingually administered PBS. (D-L) Mice sublingually administered AcHERV-triHPV at a dose of 1×10^8^ copies (D-F), 1×10^9^ copies (G-I), or 1×10^10^ copies (J-L). (M-O) Mice intramuscularly immunized with 1×10^9^ copies. Eight weeks after the first immunization, mice received vaginal challenge with luciferase-expressing PV16 (A,D,G,J,M), PV18 (B,E,H,K,N), or PV58 (C,F,I,L,O). Three days after the challenge, mice were anesthetized and injected with luciferin, and the levels of luminescence were detected with an IVIS 200 bioluminescence imaging system.

To test whether neutralizing antibody titers generated by SL administration with AcHERV-triHPV could protect mice against HPV infection, we challenged immunized mice with HPV16, -18, or -58 PVs via the vaginal route. Genital pseudo-infection with HPV16, -18, or -58 PVs was detected by monitoring the expression of the luciferase reporter gene using whole-organ, multispectral molecular imaging (Fig. 7). Non-immunized mice challenged with luciferase-encoding HPV16 PVs (Fig. 7A), HPV18 PVs (Fig. 7B) or HPV58 PVs (Fig. 7C) exhibited strong luminescence intensities, reflecting effective vaginal pseudo-infection by PVs. Mice immunized by SL administration of 1×10^8^ copies of AcHERV-triHPV showed dim luminescence in smaller spot areas after challenge with HPV16 PVs (Fig. 7D), HPV18 PVs (Fig. 7E), or HPV58 PVs (Fig. 7F). However, mice immunized by SL administration of 1×10^9^ (Fig. 7G-I) or 1×10^10^ (Fig. 7J-L) copies of AcHERV-triHPV showed no luminescence after challenge with HPV16 PVs (Fig. 7G, 7J), HPV18 PVs (Fig. 7H, 7K) or HPV58 PVs (Fig. 7I, 7L). Mice immunized by IM administration of 1×10^9^ copies of AcHERV-triHPV showed no luminescence after challenge with HPV16 PVs (Fig. 7M), HPV18 PVs (Fig. 7N), or HPV58 PVs (Fig. 7O).

## Discussion

Here, we demonstrated the humoral and cellular immunogenicity of sublingually administered AcHERV-triHPV against HPV types 16, 18, and 58. SL immunization with AcHERV-triHPV protected mice against challenge with HPV type-specific PVs. Moreover, sublingually administered AcHERV-triHPV showed a higher distribution to the lungs compared to other tissues at 15 minutes and 1 hour after administration, but did not persist in the lung for more than 24 hours.

The higher levels of AcHERV-triHPV in the lung at early time points supports the interpretation that sublingually administered AcHERV-triHPV is directly absorbed into the lung, rather than being systemically absorbed in the blood and subsequently distributed to the lung. It is likely that the systemic absorption of AcHERV-triHPV involves both paracellular and transcellular pathways across sublingual epithelial cells. It has been reported that sublingually administered antigens are transported across ductal SL epithelial cells and reach ductal antigen-presenting cells in mice [20]. In addition, it is possible that sublingually administered AcHERV-triHPV reaches the lung in part through migration of SL dendritic cells that capture the antigen. Consistent with this possibility, a recent study reported that antigen-displaying dendritic cells in the SL mucosa were detected at distant lymph nodes and in the spleen [21].

We observed the distribution of AcHERV-triHPV to the brain following SL administration. Previous studies reported that sublingually administered viral vaccines did not migrate to the brain [22, 23]. Song et al. [22] reported that live influenza A/PR/8 virus was not detected in the olfactory bulb and brain tissues, one day after SL administration. Shim et al. [23] observed that sublingually administered recombinant adenovirus encoding acute respiratory syndrome-associated coronavirus (SARS-CoV) was not detected in the olfactory bulb, concluding no redirection of the virus to the brain. Given these previous reports, it is unlikely that AcHERV-triHPV directly migrated to the brain via olfactory bulb. Rather, there exists a possibility that systemically absorbed AcHERV-triHPV might distribute to the brain passing the blood-brain barrier. Indeed, we previously observed the brain distribution of AcHERV encoding HPV16L1 following intramuscular administration [24]. In the study, the distribution levels of intramuscularly administered AcHERV encoding HPV16L1 were similar among the brain, heart, and lung tissues. Similar to our previous report [24], the brain distribution of porcine-derived adeno-associated virus was observed following intravenous injection to mice [25].

SL administration of AcHERV-triHPV induced serum IgG and vaginal IgA to an extent comparable to that of intramuscularly administered AcHERV-triHPV. Previously, we showed that SL administration of HPV16L1 protein induced humoral and cellular immune responses [26]. However, in this latter study, HPV16L1 alone was not effective in inducing immunogenicity and required co-administration of adjuvants, such as cholera toxin B, to induce prolonged and cellular immunogenicity. Although the interconnection of the mucosal and systemic immune system remains to be studied further, we can’t exclude the possibility that the type of antigens or vaccine delivery systems may affect the efficiency of mucosal immunogenicity following systemic or mucosal administration. A previous study reported that vaginal IgA immune responses against bovine papillomavirus type 1 virus-like particles were comparable following intranasal and intramuscular administration [27]. In the case of chimeric bovine papillomavirus virus-like particles incorporating sequence from human immunodeficiency virus-1 gp120, the vaginal IgA induction was higher following IM administration as compared to intravaginal administration [28]. Intramuscular administration of integrase-defective lentiviral vector carrying ovalbumin gene followed by sublingual ovalbumin administration was reported to induce persistent vaginal IgA immune responses [29]. A recent study reported that SL immunization of female human volunteers with Gardasil induced 38-fold lower serum IgG levels and 2-fold lower cervical/vaginal IgG levels than IM immunization [30], highlighting the importance of the delivery system for SL immunization.

We observed that SL or IM immunization of mice with AcHERV-triHPV induced both Th1 and Th2 immune responses (Fig. 6). Plasmid DNA vaccines have been known to induce Th1 type-specific immune responses [31]. Our observation on both Th1 and Th2 immune responses might be explained by both Th1 and Th2-stimulating adjuvant activity of AcHERV baculoviral vectors. A previous study reported the role of viral vectors in activation of T cell subsets in immune responses [32]. They observed that an adeno-associated viral (AAV) vector expressing coagulation factor IX could activate CD4+ T helper cells primarily of the Th2 subset, whereas an adenoviral vector efficiently activated coagulation factor IX-specific CTLs and T helper cells of both Th1 and Th2 subsets [32]. Baculovirus was reported to induce both Th1 and Th2 immune responses in mice following intramuscular injection, by inducing baculovirus-specific INF-γ and IL4-expressing splenocytes [33]. Intranasal immunization of a recombinant baculovirus-based vaccine expressing H7N7-hemagglutinin was shown to induce both IFN-γ and IL-4 responses in the splenocytes of the mice [34]. Thus, it is speculated that AcHERV baculoviral vector could stimulate the activation of both Th1 and Th2 cell subsets for expressed HPV antigens.

SL immunization of mice with AcHERV-triHPV completely protected against vaginal challenges with HPV type-specific PVs. It is notable that SL immunization with AcHERV-triHPV protected mice challenged via the vaginal route. Such protection against PVs indicates that SL immunization is capable of inducing levels of HPV type-specific vaginal IgA sufficient to neutralize mucosa-infecting HPV. SL administration of HPV virus-like particles were shown to induce serum-neutralizing antibodies and vaginal IgA. In the study, SL administration of HPV virus-like particles provided protection against genital challenge with HPV PVs [6]. A previous study reported that SL immunization with ovalbumin plus alpha-galactosylceramide protected mice from intravenous lung tumor challenge [35]. Another study showed that SL immunization with the β-trefoil domain of botulinum neurotoxin A heavy chain fused to adenovirus 2 fiber protein protected mice from intraperitoneal challenge with botulinum neurotoxin A [36].

Delivery of trivalent HPV via the AcHERV system induced both humoral and cellular immunogenicity in the absence of adjuvants. Several studies have sought to develop antigen delivery systems suitable for the SL route [37], including multilayered films and tablets [38]. Adjuvant-free SL vaccination may be beneficial in reducing the costs of vaccine preparation and eliminating concerns about the safety of adjuvants.

In conclusion, SL administration of AcHERV-triHPV induced both humoral and cellular immune responses to an extent comparable to that of IM administration. The induction of immunogenicity using sublingually administered AcHERV-triHPV was achieved without co-administration of any adjuvants. The extent of mucosal immunogenicity was sufficient to provide complete protection against vaginal challenge with PVs. Although this study specifically demonstrated the feasibility of using AcHERV systems for SL delivery of multivalent HPV, AcHERV systems could be used in the future to induce immunogenicity against other mucosa-infecting viruses, such as influenza and human immunodeficiency viruses.
